# Facile synthesis and characterization of Fe_3_O_4_/analcime nanocomposite for the efficient removal of Cu(II) and Cd(II) ions from aqueous media

**DOI:** 10.1186/s11671-023-03848-y

**Published:** 2023-04-25

**Authors:** Faisal K. Algethami, Asma S. Al-Wasidi, Eida S. Al-Farraj, Hanadi A. Katouah, Ehab A. Abdelrahman

**Affiliations:** 1 Department of Chemistry, College of Science, Imam Mohammad Ibn Saud Islamic University (IMSIU), Riyadh, 11623 Saudi Arabia; 2 Department of Chemistry, College of Science, Princess Nourah Bint Abdulrahman University, P.O. Box 84428, Riyadh, 11671 Saudi Arabia; 3 Department of Chemistry, Faculty of Applied Sciences, Umm Al-Qura University, Makkah, 21955 Saudi Arabia; 4 Chemistry Department, Faculty of Science, Benha University, Benha, 13518 Egypt

**Keywords:** Adsorbents, Nanostructures, Copper ions, Cadmium ions

## Abstract

In the water purification field, heavy metal pollution is a problem that causes severe risk aversion. This study aimed to examine the disposal of cadmium and copper ions from aqueous solutions by a novel Fe_3_O_4_/analcime nanocomposite. A field emission scanning electron microscope (FE-SEM), Fourier transform infrared spectroscopy (FT-IR), and X-ray diffraction were used to characterize the synthesized products. The FE-SEM images showed that the analcime and Fe_3_O_4_ samples consist of polyhedral and quasi-spherical shapes with average diameters of 923.28 and 28.57 nm, respectively. Besides, the Fe_3_O_4_/analcime nanocomposite consists of polyhedral and quasi-spherical shapes with average diameters of 1100.00 nm. The greatest uptake capability of the Fe_3_O_4_/analcime nanocomposite toward the copper and cadmium ions is 176.68 and 203.67 mg/g, respectively. The pseudo-second-order kinetic model and Langmuir equilibrium isotherm best describe the uptake of copper and cadmium ions using the Fe_3_O_4_/analcime nanocomposite. The uptake of copper and cadmium ions using the Fe_3_O_4_/analcime nanocomposite is exothermic and chemical in nature.

## Introduction

In recent decades, water contamination has been one of the greatest significant environmental problems that humans have faced. Consequently, it has garnered the interest of numerous academics and scientists [1–3]. Inorganic pollutants, also identified as soluble metallic ions, are considered one of the most important environmental issues. There is cause for concern regarding the release of toxic metals into water bodies. Metal ions, for example, Pb(II), Cr(III), Hg(II), Cd(II), Zn(II), Co(II), and Ni(II) are known to be poisonous when they discharged into the environment in amounts that pose significant human health risks [4–6]. These metals frequently accumulate in various organs and tissues of living things, causing a variety of impairments and diseases. In the removal of heavy metals, numerous methods have been used and recognized. Among these methods are photocatalytic degradation, electrochemical precipitation, ion exchange, membrane separation, and adsorption [7–11]. Nevertheless, these conventional methods have limitations, such as sensitivity to operating environments, low efficiency, high disposal costs, and sludge production. Adsorption has been introduced in recent years as a promising technique due to its ease of application, high stability and efficiency, practicability, and economic advantages [12–18]. The adsorption method is widely used by scientists to remove various kinds of contaminants from water and wastewater. Mehdizadeh et al. synthesized magnetic nanocomposite for the removal of methylene blue dye from aqueous solutions where the maximum uptake capability equals 16.129 mg/g. In the first step, magnetite nanoparticles were synthesized using the co-precipitation method then modified with 3-aminopropyltriethoxysilane and acryloyl chloride, respectively. The final nanocomposite was created by grafting itaconic acid and 2-hydroxyethyl methacrylate onto modified nanoparticles via in situ copolymerization [19]. Also, iron-based metal–organic framework and poly(aniline-co-pyrrole) nanospheres were utilized as novel adsorbents for the removal of various kinds of organic and inorganic pollutants from aqueous media [20, 21]. Analcime is a type of sodium aluminum silicate (i.e. zeolites) whose structure is based on (Si, Al)O_4_ tetrahedra sharing corners, which have pores, channels, and/or cavities at the molecular level [22]. The crystal lattice of zeolites (such as analcime) is formed by substituting some tetravalent silicon ions with trivalent aluminum ions. The substitution process results in a net negative charge that is neutralized by positive sodium ions, which can do ion exchange with other ions such as cadmium and copper. Hence, it is expected that the analcime will be used to treat water pollution through the ion exchange process [23]. In addition, nano magnetic oxides such as Fe_2_O_3_ and Fe_3_O_4_ have been widely used in water purification due to their high surface area, superparamagnetic properties, simple synthesis process, appropriate biocompatibility, and non-toxicity [24–28]. Also, it contains hydroxyl groups that have the ability to do ion exchange with other ions. Naini et al. synthesized Fe_3_O_4_/SiO_2_ composite for the uptake of palladium ions from aqueous solutions where the maximum uptake capability equals 1.35 mg/g [25]. Kumar et al. synthesized Fe_2_O_3_/CdSe nanocomposite for the uptake and detection of picric acid. The quenching constant of nanocomposite with picric acid was 4.30 × 10^4^ M^−1^ in DMSO with a limit of detection up to 2.20 μM as a consequence of turn off sensing [26]. Wang et al. synthesized g-C_3_N_4_/Fe_2_O_3_ nanocomposite for photocatalytic removal of indoor formaldehyde under visible light [27]. Wang et al. synthesized Fe_2_O_3_/HNb_3_O_8_ nanocomposite for the uptake of ethyl mercaptan in methane gas where the maximum uptake capability equals 48.05 mg/g [28]. Cadmium poisoning occurs as a result of consuming drinks or food polluted with large concentrations of it, and diseases of cadmium poisoning appear after several years and after the accumulation of large quantities of it in the body. In addition, among the main symptoms of cadmium poisoning; vomiting, nausea, and abdominal pain. Besides, cadmium has toxic influences on the skeletal system owing to its impact on phosphorus and calcium metabolism, where a decrease in calcium absorption occurs and the result is osteoporosis. Cadmium has a direct impact on high blood pressure and kidney function disorder [29]. Consumption of even relatively small amounts of copper may cause nausea, vomiting, and diarrhea, while consumption of large amounts can damage the kidneys, inhibit urine production, cause anemia due to the rupture of red blood cells (hemolytic anemia), and even death [30]. Many adsorbents are used to remove cadmium and copper ions. Pereira et al. synthesized aminated cellulose as a versatile adsorbent for the disposal of copper ions from aqueous solutions where the maximum uptake capability equals 69.27 mg/g [31]. Deng et al. synthesized aminated resin by surface-initiated atom transfer radical polymerization and subsequent amination reaction for the disposal of copper ions from aqueous solutions. The maximum uptake capability of the adsorbent towards copper ions equals 139.80 mg/g [32]. Kuang et al. synthesized chitin/chitosan-based aerogel for the disposal of copper ions from aqueous solutions where the maximum uptake capability equals 59.21 mg/g [33]. Yang et al. synthesized graphene oxide and carboxylated graphene oxide for the disposal of copper ions from aqueous solutions where the maximum uptake capability equals 55.47 and 62.02 mg/g, respectively [34]. Alvarez-Alvarez et al. synthesized alginate-halloysite for the disposal of cadmium ions from aqueous solutions where the maximum uptake capability equals 88.00 mg/g [35]. Zhao et al. synthesized goethite-modified montmorillonite for the disposal of cadmium ions from aqueous solutions where the maximum uptake capability equals 50.61 mg/g [36]. Joseph et al. synthesized FAU zeolite for the disposal of cadmium ions from aqueous solutions where the maximum uptake capability equals 74.07 mg/g [37]. The objective of this work was to evaluate the ability of Fe_3_O_4_/analcime nanocomposites to remove copper and cadmium ions. The feature of Fe_3_O_4_/analcime nanocomposite is its ability to remove copper and cadmium ions using the ion exchange adsorption process, and hence the adsorption capacity is expected to be great. The FE-SEM, XRD, and FT-IR techniques were used to illustrate the structural and physical properties of the synthesized adsorbent. Moreover, the effects of temperature, pH, time, and concentration on the uptake of copper and cadmium ions from aqueous media were examined.

## Experimental

### Chemicals

Iron(II) sulfate heptahydrate (FeSO_4_·7H_2_O), sodium hydroxide (NaOH), iron(III) chloride hexahydrate (FeCl_3_·6H_2_O), aluminum chloride hexahydrate (AlCl_3_·6H_2_O), cadmium(II) nitrate tetrahydrate (Cd(NO_3_)_2_·4H_2_O), glutamine (C_5_H_10_N_2_O_3_), copper sulfate (CuSO_4_), hydrochloric acid (HCl), ammonium hydroxide (NH_4_OH), potassium nitrate (KNO_3_), ethylenediaminetetraacetic acid disodium salt dihydrate (C_10_H_14_N_2_Na_2_O_8_·2H_2_O), and fumed silica (SiO_2_) were obtained from the Sigma Aldrich Chemical Company then utilized without purifying. Besides, the analcime was prepared according to the procedure described by Youssef et al. [38]. Moreover, Fe_3_O_4_ was prepared according to the procedure described by Kang et al. [39].

### Synthesis of Fe_3_O_4_/analcime nanocomposite

1.50 g of the Fe_3_O_4_ and 7.50 g of analcime were refluxed under stirring for 24 h utilizing 100 mL of ethanol. Then, a magnet was used for removing the formed nanocomposite then the product was washed multiple times with distilled water and dried in an oven.

### Characterization

The X-ray diffraction patterns of the chemically synthesized powders were gotten utilizing a D8 Advance X-ray diffractometer with a copper anode (λ of CuK_α_ = 1.5 Å). FT-IR spectra of the synthesized powders were gotten in the range from 4000 to 400 cm^−1^ using a Nicolet iS50 spectrophotometer pressed into potassium bromide. The morphology of the synthesized powders was investigated using a field emission scanning electron microscopy of model JSM-IT800 Schottky. The concentrations of copper and cadmium ions were estimated utilizing a Perkin Elmer-3300 multi-element atomic absorption spectrometer. The magnetic properties of the synthesized composite were determined at room temperature (27 °C) using a vibrating sample magnetometer (VSM-Cryogenic Limited PPMS). The stability of the synthesized composite was studied under nitrogen atmosphere using a thermal gravimetric analyzer (TGA-Shimadzu DT-60H) with a heating speed of 10 °C/min.

### Disposal of copper and cadmium ions from aqueous solutions

The batch adsorption method was used to evaluate the adsorption processes of copper and cadmium ions utilizing the Fe_3_O_4_/analcime nanocomposite. In this regard, 0.05 g of the Fe_3_O_4_/analcime nanocomposite was added to 50.00 mL of about 200.00 mg/L of cadmium or copper solution. After that, the resulting mixture was magnetically stirred for a definite time. Finally, the adsorbent was separated using centrifugation then the concentration of copper or cadmium ions in the filtrate was determined at 193.70 and 324.70 nm using a Perkin Elmer-3300 multi-element atomic absorption spectrometer, respectively. Several factors affecting adsorption processes were studied, such as pH (2.50–8.00), time (5.00–120.00 min), uptake temperature (298.00–328.00 K), and concentration (100.00–300.00 mg/L).

The uptake percentage of the copper or cadmium ions (% R) and the uptake capability of the Fe_3_O_4_/analcime nanocomposite (Q) were determined using Eqs. 1 and 2, respectively.

1 $$\% {\text{R}} = \frac{{C_{o} - C_{eq} }}{{C_{o} }} \times 100$$

2 $$Q = (C_{o} - C_{eq} ) \times \frac{V}{M}$$

where, C_o_ is the initial concentration of the cadmium or copper ions (mg/L), C_eq_ is the equilibrium concentration of the cadmium or copper ions (mg/L) and m is the dry mass of the Fe_3_O_4_/analcime nanocomposite (g). Besides, V is considered the taken volume of the cadmium or copper solution (L).

The point of zero charge (pH_PZC_) of the Fe_3_O_4_/analcime nanocomposite was estimated, as illustrated by Khalifa et al., as the following [40]: Separately, 0.25 g of the Fe_3_O_4_/analcime nanocomposite were mixed with 70.00 mL of about 0.03 M KNO_3_ solutions. Moreover, the primary pH (pH_i_) of the KNO_3_ solutions was examined in the range from 2.00 to 12.00. Each adsorbent/KNO_3_ blend was magnetically stirred for 9 h. The final pH values (pH_f_) were measured and plotted against the initial pH values (pH_i_). The pH_pzc_ is the pH_f_ level where a characteristic plateau was obtained.

### Regeneration and reusability of the Fe_3_O_4_/analcime nanocomposite

The adsorption was carried out as the following; 0.05 g of the Fe_3_O_4_/analcime nanocomposite was added to 50.00 mL of about 200.00 mg/L (pH = 6.50) of cadmium or copper solution. After that, the resulting mixture was magnetically stirred for 80 min. Finally, the nanocomposite was separated using centrifugation then the concentration of copper or cadmium ions in the filtrate was determined at 193.70 and 324.70 nm using a Perkin Elmer-3300 multi-element atomic absorption spectrometer, respectively. To regenerate the nanocomposite, loaded copper or cadmium ions is completely desorbed from the surface of the nanocomposite by stirring the nanocomposite loaded with copper or cadmium ions in 50 mL of 1 M ethylenediaminetetraacetic acid disodium salt dihydrate for 30 min. Afterward, the regenerated nanocomposite was used for the removal of copper and cadmium ions for four consecutive cycles as previously described.

## Results and discussion

### Identification of the synthesized powders

**Fig. 1:**
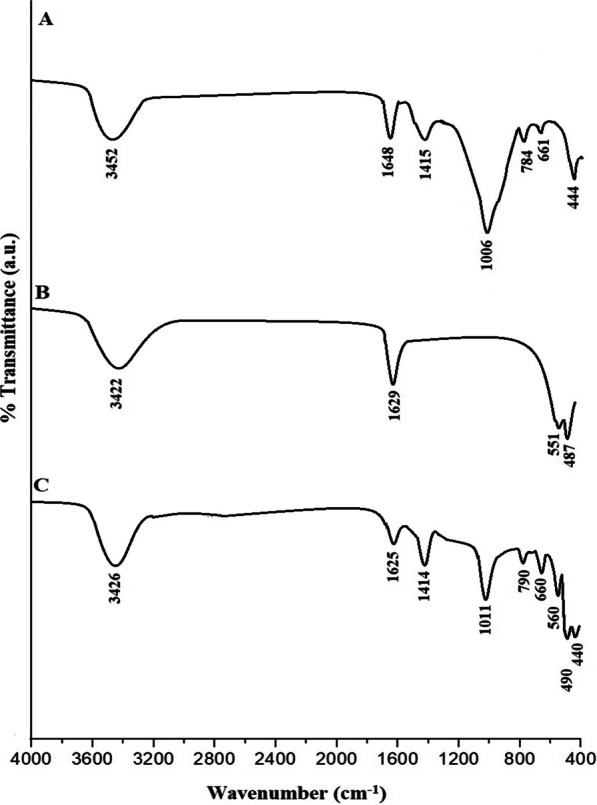
The FT-IR spectra of the analcime (**A**), Fe_3_O_4_ (**B**), and Fe_3_O_4_/analcime nanocomposite (**C**)

Figure 1A–C displays the FT-IR spectra of the analcime, Fe_3_O_4_, and Fe_3_O_4_/analcime nanocomposite, respectively. Moreover, the FT-IR bands of the analcime, which were appeared at 444, 661, and 784 cm^−1^, are due to the bending vibrations, internal symmetric vibrations, and external symmetric vibrations of W–O–W (W=Si and/or Al), respectively. Also, the FT-IR of the analcime, which were appeared at 1006, 1415, 1648, and 3452 cm^−1^, are because of the internal asymmetric vibrations of W–O–W, external asymmetric vibrations of W–O–W, bending vibrations of H–O–H, and stretching vibrations of H–O–H, respectively. The FT-IR bands of the Fe_3_O_4_, which were appeared at 487 and 551 cm^−1^, are because of the stretching vibrations of Fe–O. Furthermore, the FT-IR bands of the Fe_3_O_4_, which were appeared at 1629 and 3422 cm^−1^, are due to the bending and stretching vibrations of H–O–H, respectively. The FT-IR bands of the analcime in the Fe_3_O_4_/analcime nanocomposite were observed at 440, 660, 790, 1011, 1414, 1625, and 3426 cm^−1^. Moreover, the FT-IR bands of the Fe_3_O_4_ in the Fe_3_O_4_/analcime nanocomposite were observed at 490 and 560 cm^−1^ [40–43]. Comparing the FT-IR spectra of analcime, Fe_3_O_4_, and Fe_3_O_4_/analcime, the results displayed that almost all the bands of the analcime and Fe_3_O_4_ were observed in the synthesized Fe_3_O_4_/analcime nanocomposite. Hence, this indicating the successful synthesis of Fe_3_O_4_ modified with analcime.

**Fig. 2:**
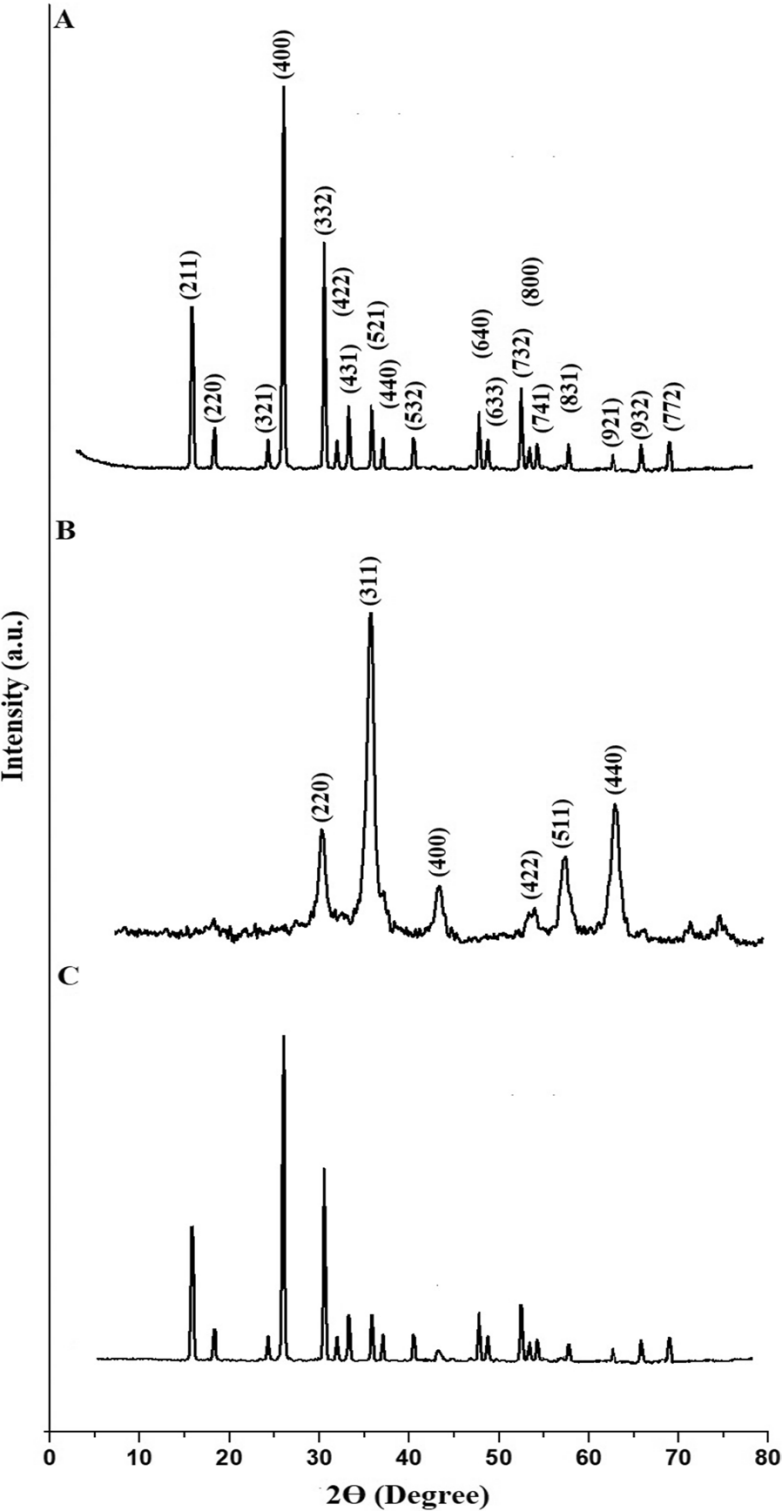
The XRD patterns of the analcime (**A**), Fe_3_O_4_ (**B**), and Fe_3_O_4_/analcime nanocomposite (**C**)

Figure 2A–C displays the XRD patterns of the analcime, Fe_3_O_4_, and Fe_3_O_4_/analcime nanocomposite, respectively. In addition, the XRD peaks, which appeared at 2Ɵ = 15.83°, 18.39°, 24.33°, 25.99°, 30.64°, 31.90°, 33.25°, 35.88°, 37.03°, 40.49°, 47.84°, 48.68°, 52.46°, 53.29°, 54.25°, 57.71°, 62.75°, 65.90°, and 69.04°, are due to the (211), (220), (321), (400), (332), (422), (431), (521), (440), (532), (640), (633), (732), (800), (741), (831), (921), (932), and (772) miller indices of the analcime (Na(AlSi_2_O_6_)(H_2_O)) as clarified from JCPDS No. 01-070-1575. The XRD peaks, which appeared at 2Ɵ = 30.22°, 35.57°, 43.23°, 53.83°, 57.29°, and 62.85°, are due to the (220), (311), (400), (422), (511), and (440) miller indices of the Fe_3_O_4_ as clarified from JCPDS No. 85-1436. Also, the intensity of the XRD peaks of the Fe_3_O_4_/analcime nanocomposite was affected, but it is similar to that of the analcime. In the XRD pattern of nanocomposite, the most intense peak of Fe_3_O_4_ at 2Ɵ = 35.57° was found to be diminished due to combination of Fe_3_O_4_ with analcime that have more intense peaks than Fe_3_O_4_. The mean crystallite sizes of the analcime, Fe_3_O_4_, and Fe_3_O_4_/analcime nanocomposite, which were determined using Scherrer equation, are 80.24, 15.27, and 98.29 nm, respectively [38].

**Fig. 3:**
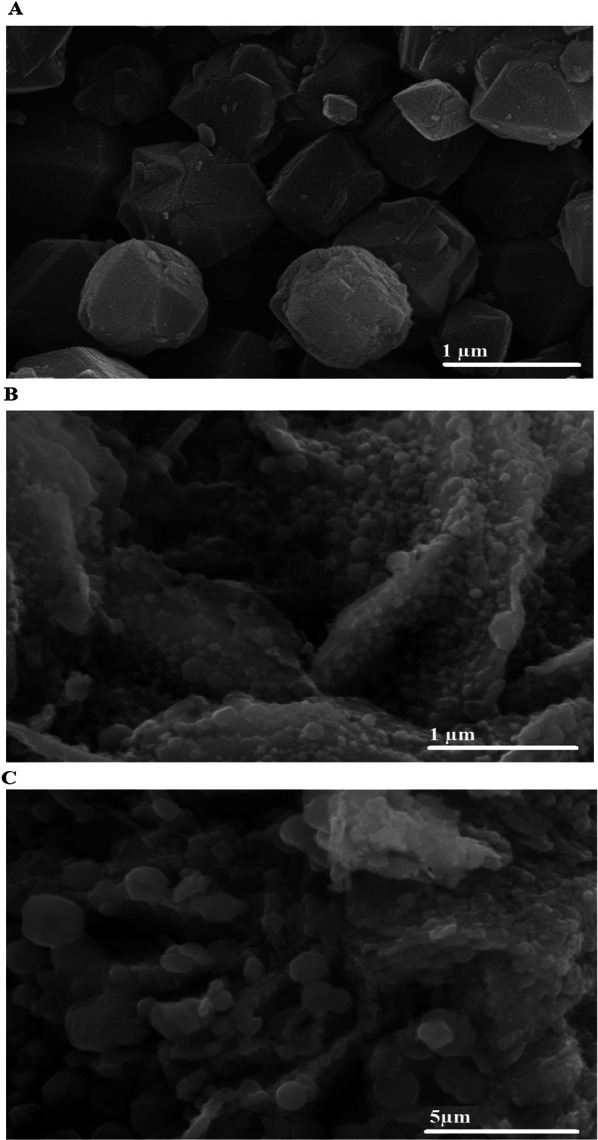
The FE-SEM morphological images of the analcime (**A**), Fe_3_O_4_ (**B**), and Fe_3_O_4_/analcime nanocomposite (**C**)

Figure 3A–C represents the FE-SEM morphological images of the analcime, Fe_3_O_4_, and Fe_3_O_4_/analcime nanocomposite, respectively. In addition, the results showed that the analcime and Fe_3_O_4_ samples consist of polyhedral and quasi-spherical shapes with average diameters of 923.28 and 28.57 nm, respectively. Besides, the Fe_3_O_4_/analcime nanocomposite consists of polyhedral and quasi-spherical shapes with average diameters of 1100.00 nm. Upon incorporation of the Fe_3_O_4_ nanoparticles into the analcime framework, a distortion of the analcime's original polyhedral structure was observed. This suggests a bonding interaction between analcime and Fe_3_O_4_ nanoparticles, resulting in morphological changes. It is known that the SEM shows the shape of the surface of the samples, not the shape of the particles, which could be a combination of multiple particles. Therefore, SEM cannot be used to determine whether the sample is nanosized or not. XRD determines whether the sample is nanosized or not.

**Fig. 4:**
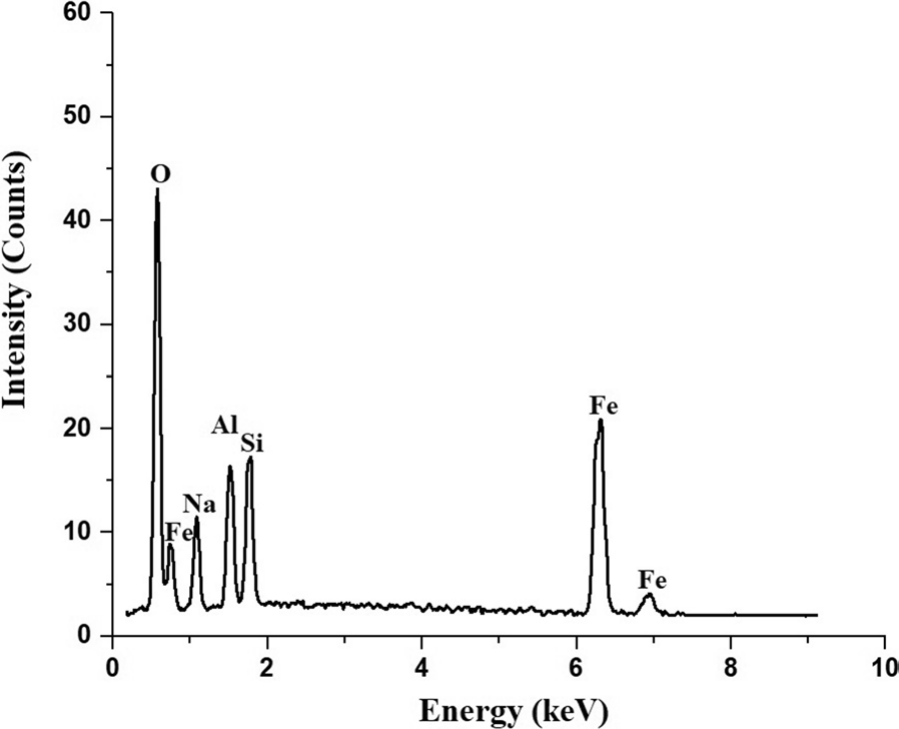
The EDX pattern of the Fe_3_O_4_/analcime nanocomposite

Figure 4 represents the EDX pattern of the Fe_3_O_4_/analcime nanocomposite. The results displayed that the Fe_3_O_4_/analcime nanocomposite consists of Fe, Si, Al, O, and Na with weight percentages equal to 18.76, 12.82, 12.57, 51.10, and 4.75%, respectively. Hence, this is a confirmation that there is a bonding interaction between analcime and Fe_3_O_4_ nanoparticles.

**Fig. 5:**
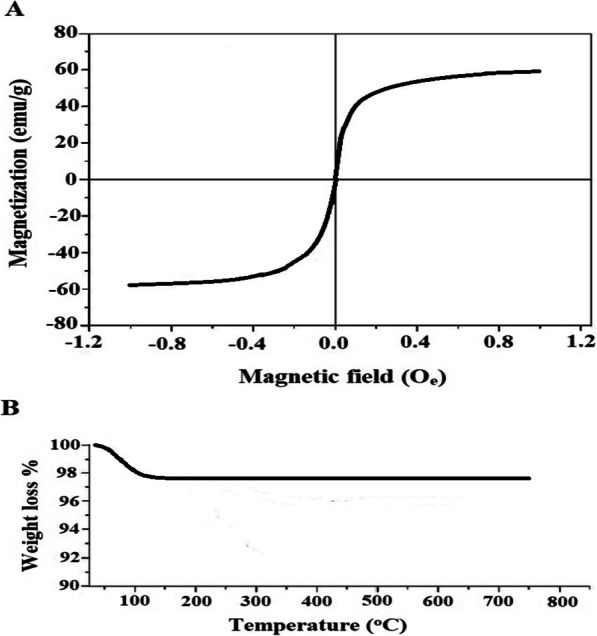
VSM (**A**) and TGA (**B**) curves of the synthesized nanocomposite

The plot of magnetization of the synthesized nanocomposite at 27 °C versus magnetic field is displayed in Fig. 5A. The plot indicates that the synthesized nanocomposite exhibits superparamagnetic behavior with zero coercivity and remanence. The saturation magnetization (MS) is around 59.50 emu/g. In order to determine the stability of the synthesized nanocomposite, thermal gravimetric analysis (TGA) has been carried out as shown in Fig. 5B. The results confirmed that there is about 2.43% weight loss due to the removal of residual moisture owing to the adsorbed water molecules. Hence, this confirms the stability of the synthesized nanocomposite.

### Disposal of cadmium and copper ions from aqueous solutions

#### Effect of pH

**Fig. 6:**
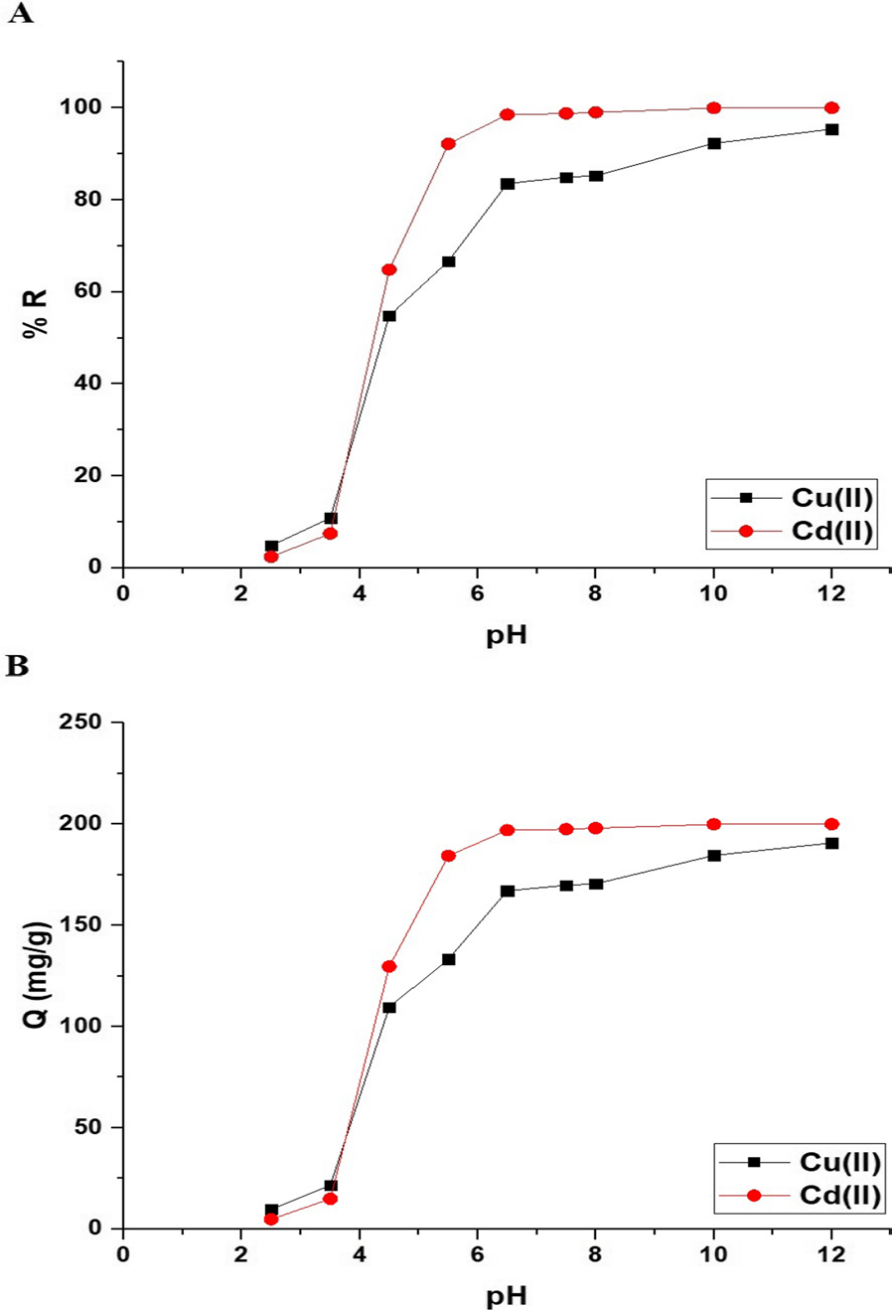
The influence of solution pH on the uptake percentage of cadmium and copper ions (**A**) and the uptake capability of the Fe_3_O_4_/analcime nanocomposite (**B**)

**Fig. 7:**
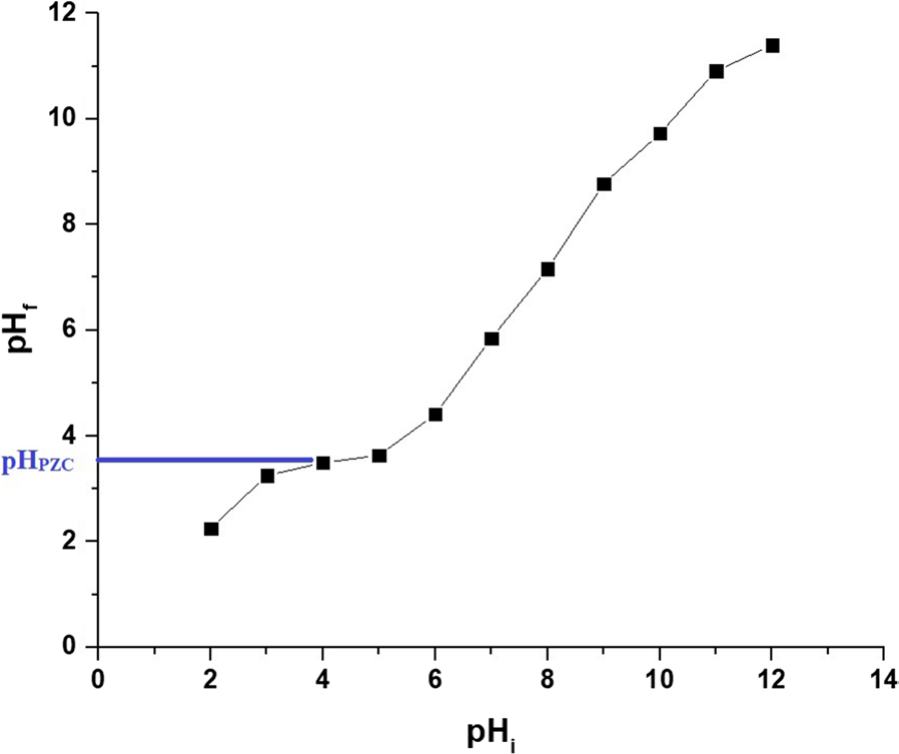
The point of zero charge of the Fe_3_O_4_/analcime nanocomposite

Figure 6A and B represents the impact of pH on the uptake percentage of copper and cadmium ions and the uptake capability of the Fe_3_O_4_/analcime nanocomposite, respectively. The results showed that both the uptake percentage and the uptake capability increased significantly with the increase of the pH from 2.50 to 6.50. In addition, the increase was very slight when the pH was increased from 6.50 to 8.00 owing to the constant amount of the active sites of the adsorbent. Therefore, pH 6.50 is the ideal value at which the highest uptake rate of copper and cadmium ions is achieved. At pH 6.50, the uptake percentage of copper and cadmium ions using the Fe_3_O_4_/analcime nanocomposite is 85.56 and 98.50%, respectively. Besides, the greatest uptake capability of the Fe_3_O_4_/analcime nanocomposite toward copper and cadmium ions is 167.11 and 197.00 mg/g, respectively. To understand the influence of pH on the adsorption processes of the cadmium and copper ions, the point of zero charge (pH_PZC_) of the Fe_3_O_4_/analcime nanocomposite was determined and found to be 3.40, as shown in Fig. 7. At low pH values (i.e. pH < pH_PZC_*)*, the number of positively charged sites of the Fe_3_O_4_/analcime nanocomposite increased, which did not favor the uptake of positively charged copper or cadmium ions owing to electrostatic repulsion [44, 45].At higher pH values (i.e. pH > pH_PZC_*)*, the number of negatively charged sites of the Fe_3_O_4_/analcime nanocomposite increased, which favor the uptake of positively charged copper or cadmium ions owing to electrostatic attraction [44, 45]. At higher pH values (in the range from 10 to 12), the precipitation of Cu(II) and Cd(II) ions with hydroxides is another reason for high removal.

#### Effect of time

**Fig. 8:**
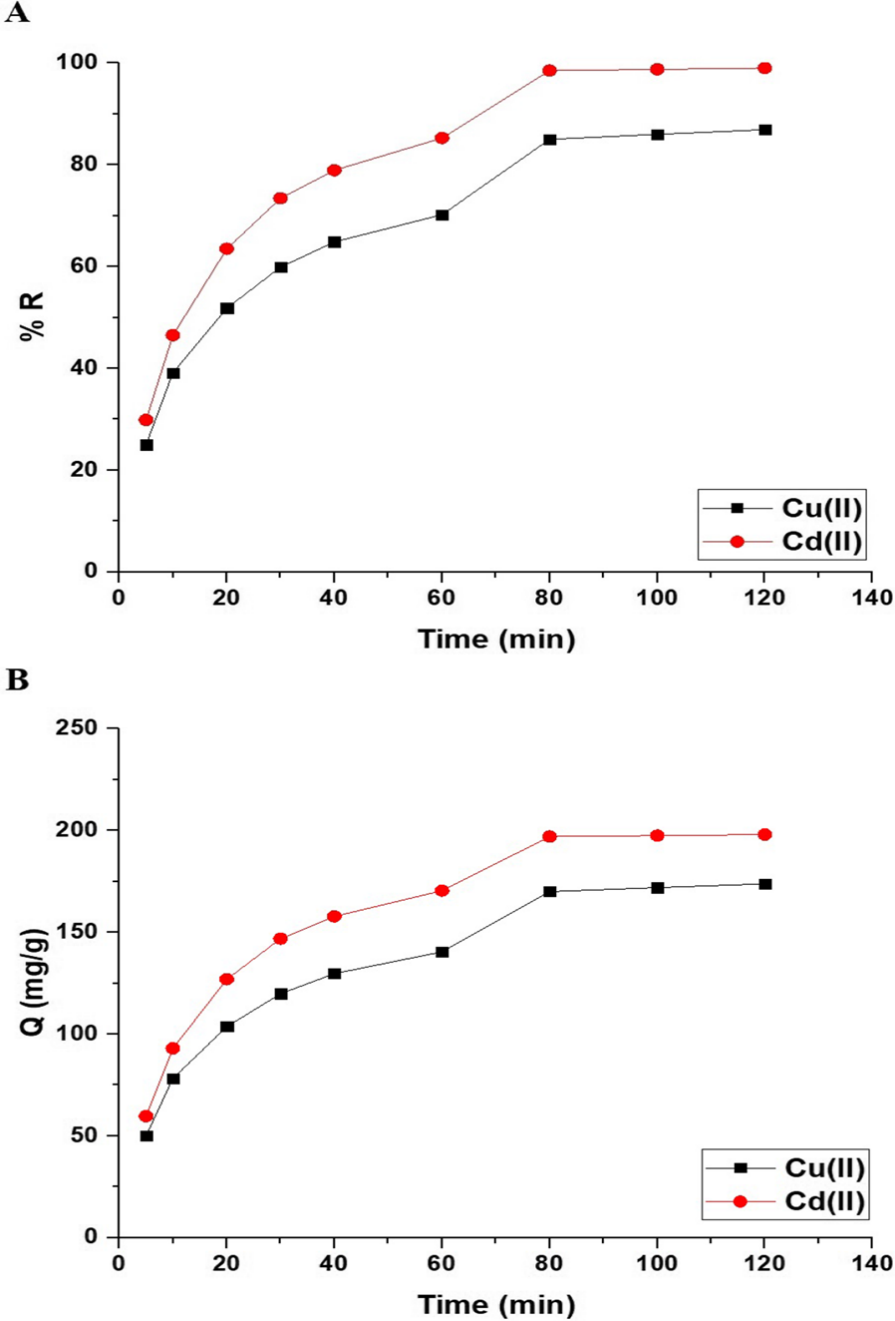
The influence of disposal time on the uptake percentage of cadmium and copper ions (**A**) and the uptake capability of the Fe_3_O_4_/analcime nanocomposite (**B**)

**Fig. 9:**
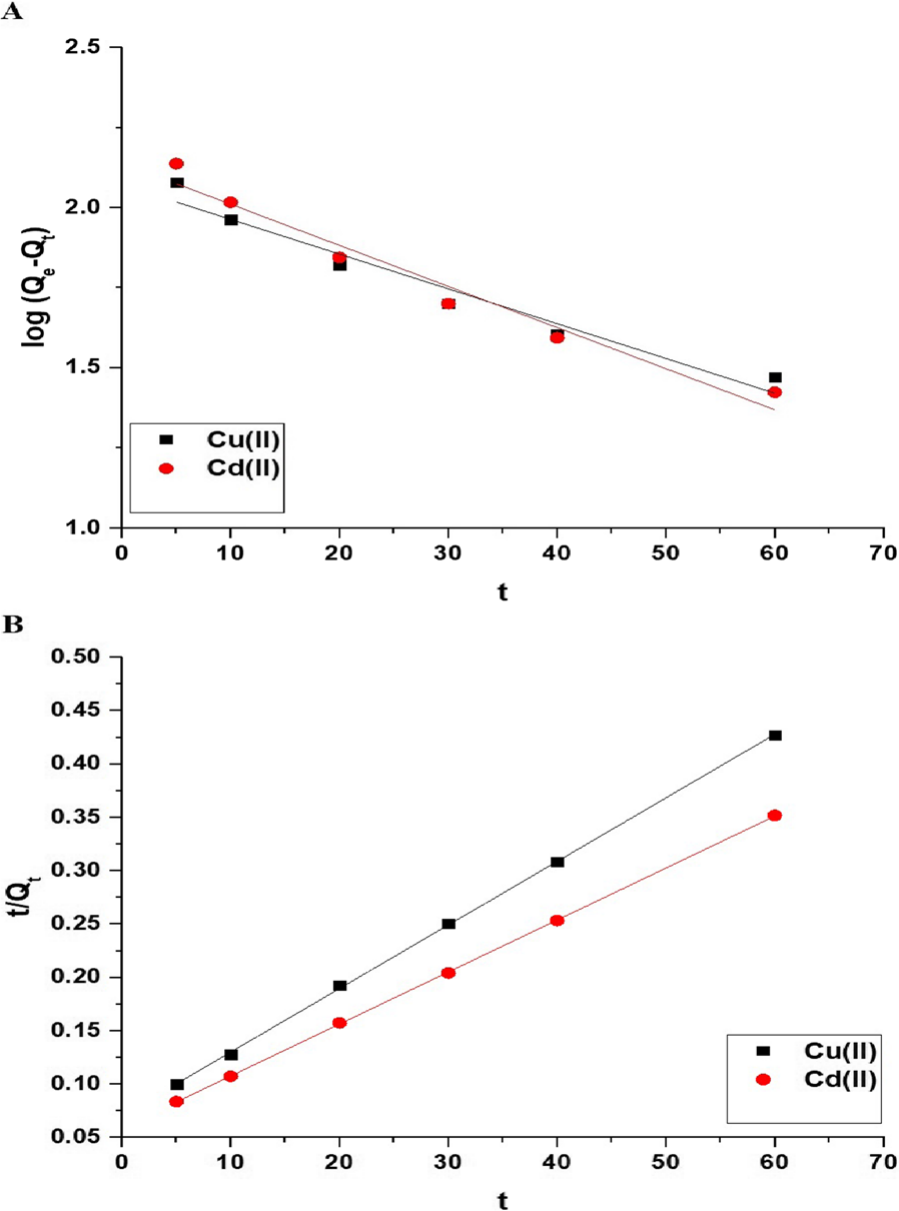
The applied pseudo-first-order (**A**) and pseudo-second-order (**B**) kinetic models for the uptake of cadmium and copper ions by the Fe_3_O_4_/analcime nanocomposite

Figure 8A and B exhibits the impact of disposal time on the uptake percentage of copper and cadmium ions and the uptake capability of the Fe_3_O_4_/analcime nanocomposite, respectively. The results showed that both the uptake percentage and the uptake capability increased significantly with the increase of the time from 5.00 to 80.00 min. In addition, the increase was very slight when the disposal time was changed from 80.00 to 120.00 min due to the saturation of the active sites of the adsorbent. Therefore, 80.00 min is the ideal value at which the greatest uptake rate of copper and cadmium ions is achieved. At time equals 80.00 min, the uptake percentage of copper and cadmium ions utilizing the Fe_3_O_4_/analcime nanocomposite is 85.00 and 98.50%, respectively. Besides, the greatest uptake capability of the Fe_3_O_4_/analcime nanocomposite toward copper and cadmium ions is 170.00 and 197.00 mg/g, respectively. Two kinetic models, pseudo-first-order (Eq. 3) and pseudo-second-order (Eq. 4), were applied to the experimental data in order to comprehend the adsorption processes of copper and cadmium ions using the Fe_3_O_4_/analcime nanocomposite, as shown in Fig. 9A and B, respectively [44, 45].

3 $$\log \left( {Q_{e} - Q_{t} } \right) = \log Q_{e} - \frac{{k_{1} }}{2.303}t$$

4 $$\frac{t}{{Q_{t} }} = \frac{1}{{k_{2} Q_{e}^{2} }} + \frac{1}{{Q_{e} }}t$$

where, Q_t_ is the amount of copper or cadmium ions adsorbed at time t (mg/g), Q_e_ is the quantity of copper or cadmium ions adsorbed at equilibrium (mg/g), k_1_ is the pseudo-first-order rate constant (1/min), and k_2_ is the pseudo-second-order rate constant (g/mg.min). The kinetic constants were presented in Table 1. The pseudo-first-order model provided poor fitting with low correlation coefficient values (R^2^) compared with the pseudo-second-order model. Consequently, the pseudo-second-order model may best describe the uptake of copper or cadmium ions using the Fe_3_O_4_/analcime nanocomposite.

**Table 1:** The kinetic constants for the uptake of copper and cadmium ions by the Fe_3_O_4_/analcime nanocomposite

Metal ion	Pseudo-first-order			Pseudo-second-order		
	Q_e_ (mg/g)	k_1_ (1/min)	R^2^	Q_e_ (mg/g)	k_2_ (g/mg.min)	R^2^
Cu(II)	118.02	0.0250	0.9485	167.78	0.0005	0.9972
Cd(II)	137.76	0.0296	0.9569	205.34	0.0004	0.9993

#### Effect of temperature

**Fig. 10:**
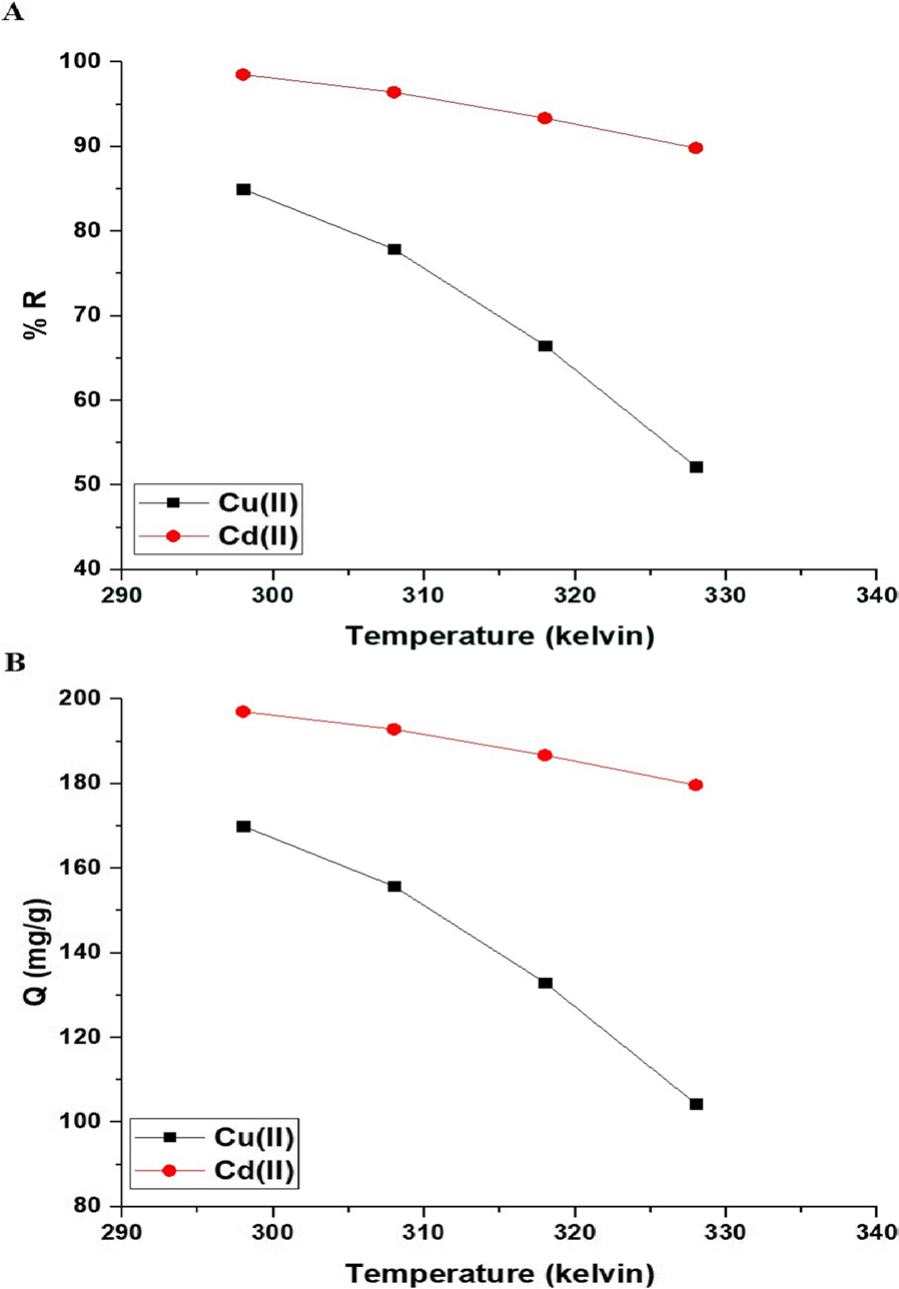
The influence of uptake temperature on the uptake percentage of cadmium and copper ions (**A**) and the uptake capability of the Fe_3_O_4_/analcime nanocomposite (**B**)

**Fig. 11:**
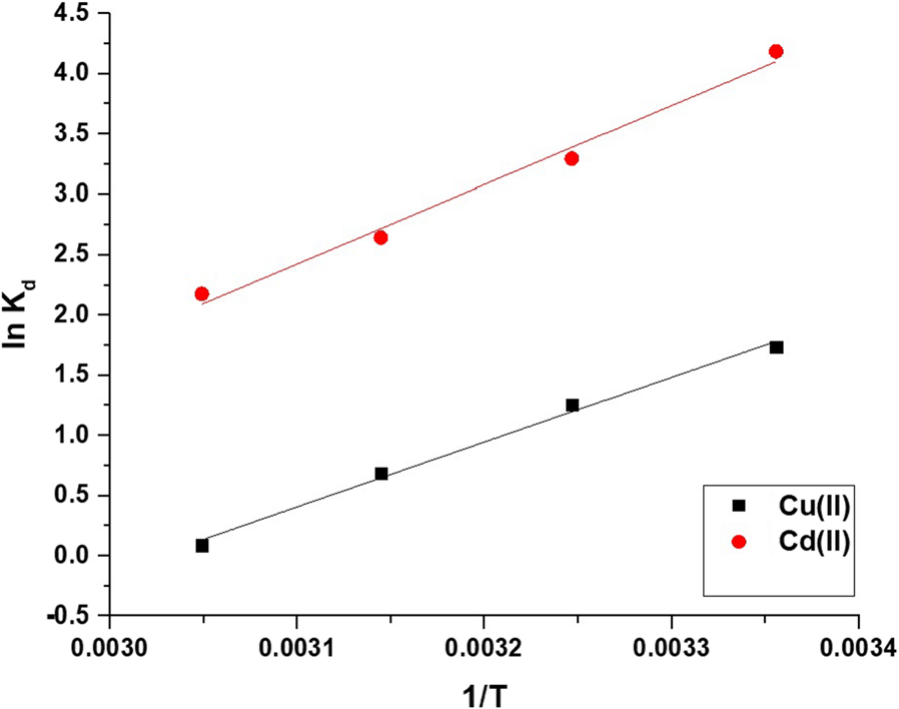
The relation between ln K_d_ and 1/T for the uptake of copper and cadmium ions by the Fe_3_O_4_/analcime nanocomposite

Figure 10A and B represents the impact of uptake temperature on the uptake percentage of cadmium and copper ions and the uptake capability of the Fe_3_O_4_/analcime nanocomposite, respectively. The results showed that both the uptake percentage and the uptake capability decreased significantly with the increase of the temperature from 298.00 to 328.00 K. Therefore, 298.00 K is the ideal value at which the greatest uptake rate of copper and cadmium ions is achieved. Exploiting Eqs. 5, 6, and 7, the thermodynamic parameters, for example, change in free energy (△G^o^, KJ/mol), change in enthalpy (△H^o^, KJ/mol), and change in entropy (△S^o^, KJ/molK), can be determined [44, 45]. The plots of ln K_d_ versus 1/T were presented in Fig. 11. The kinetic constants were presented in Table 2.

5 $$\ln K_{d} = \frac{{\Delta S^{o} }}{R} - \frac{{\Delta H^{o} }}{RT}$$

6 $$\Delta G^{o} = \Delta H^{o} - T\Delta S^{o}$$

7 $$K_{d} = \frac{{Q_{e} }}{{C_{eq} }}$$

where, K_d_ is the distribution constant (L/g) whereas R is the universal gas constant (KJ/mol kelvin). T is the uptake temperature (kelvin). The uptake of cadmium and copper ions using the Fe_3_O_4_/analcime nanocomposite is exothermic, as revealed by the negative sign of △H^o^ values. Besides, the values of △H^o^ are higher than 40 kJ/mol, revealing that the adsorption is chemical in nature. Entropy is a measure of randomness, and its positive value in the case of the disposal of copper and cadmium ions using the Fe_3_O_4_/analcime nanocomposite means that these ions move randomly in the solution and go to the adsorbent from all directions, and thus the separation efficiency increases. The negative values of △G^o^ revealed that the uptake of copper and cadmium ions occurred spontaneously.

**Table 2:** The values of △H^o^, △G^o^, and △S^o^ for the disposal of cadmium and copper ions by the Fe_3_O_4_/analcime nanocomposite

Metal ion	△H^o^ (KJ/mol)	△S^o^ (KJ/molK)	△G^o^ (KJ/mol)			
			298	308	318	328
Cu(II)	− 44.98	0.1360	− 85.53	− 86.89	− 88.25	− 89.61
Cd(II)	− 54.69	0.1493	− 99.18	− 100.67	− 102.16	− 103.65

#### Effect of concentration

**Fig. 12:**
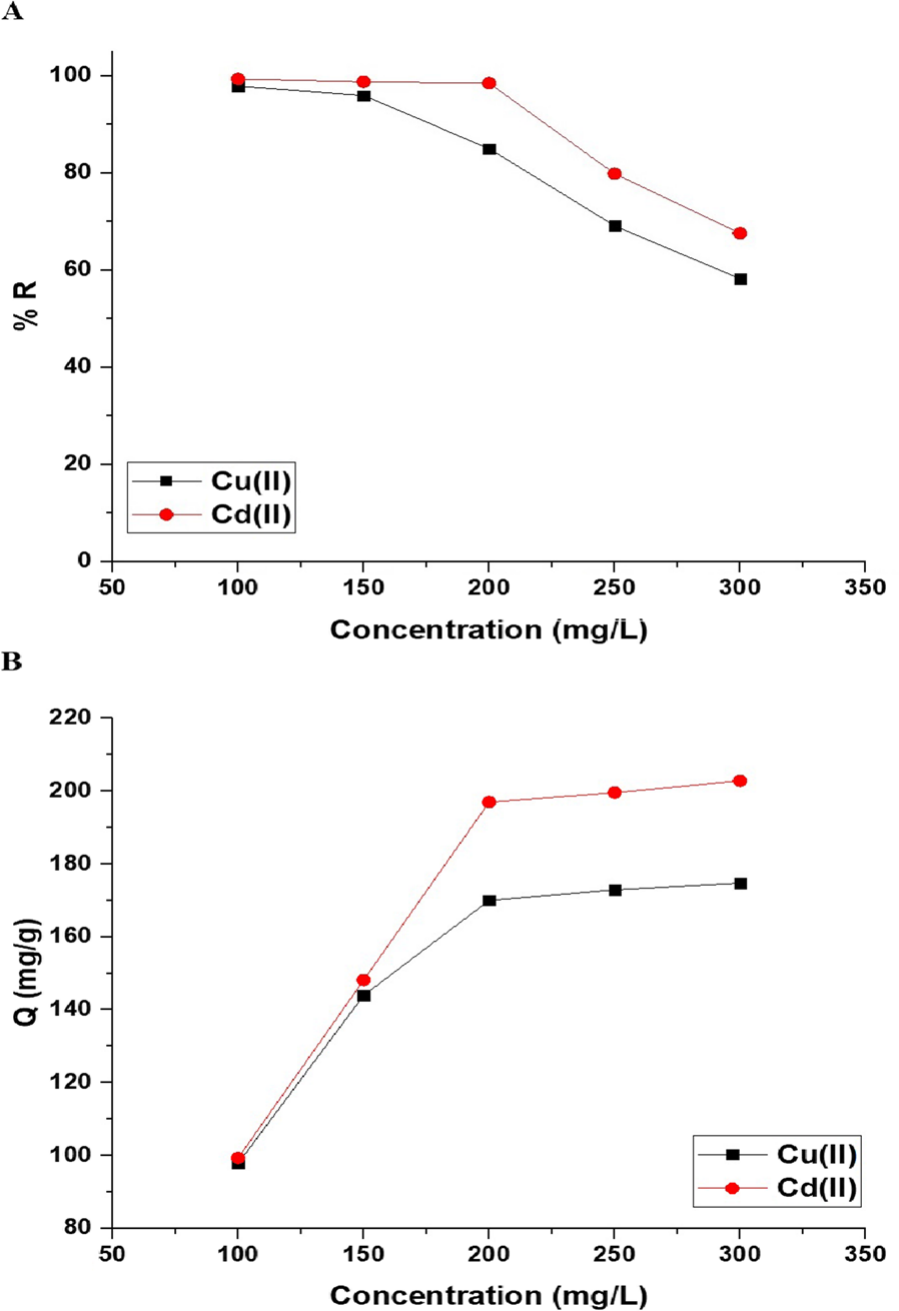
The effect of concentration on the uptake percentage of copper and cadmium ions (**A**) and the uptake capability of the Fe_3_O_4_/analcime nanocomposite (**B**)

**Fig. 13:**
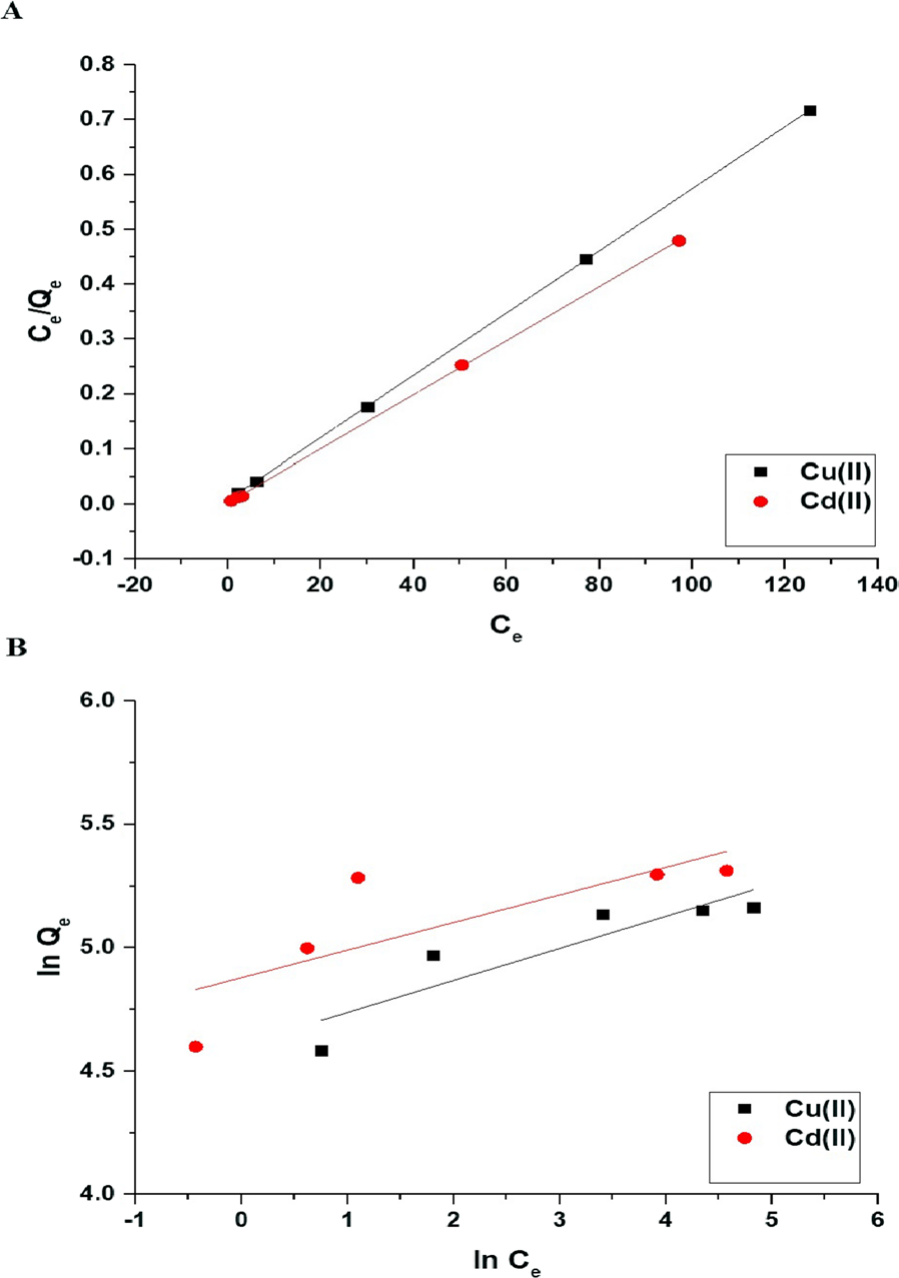
The applied Langmuir (**A**) and Freundlich (**B**) equilibrium isotherms for the uptake of copper and cadmium ions by the Fe_3_O_4_/analcime nanocomposite (**B**)

Figure 12A and B represents the impact of concentration on the uptake percentage of copper and cadmium ions and the uptake capability of the Fe_3_O_4_/analcime nanocomposite, respectively. The results showed that the uptake percentage decreased significantly whereas the uptake capability increased significantly with the increase of the concentration from 100.00 to 300.00 mg/L. Two equilibrium isotherms, Langmuir (Eq. 8) and Freundlich (Eq. 9), were applied to the experimental data in order to comprehend the adsorption processes of copper and cadmium ions using the Fe_3_O_4_/analcime nanocomposite, as shown in Fig. 13A and B, respectively [44, 45].

8 $$\frac{{C_{eq} }}{{Q_{e} }} = \frac{1}{{k_{L} Q_{\max } }} + \frac{{C_{eq} }}{{Q_{\max } }}$$

9 $$\ln Q_{e} = \ln k_{F} + \frac{1}{n}\ln C_{eq}$$

where, 1/n is the heterogeneity constant whereas k_L_ is the equilibrium constant of the Langmuir isotherm (L/mg). In addition, k_F_ is the equilibrium constant of the Freundlich isotherm (mg/g)(L/mg)^1/n^ whereas Q_max_ is the maximum uptake capability of the Langmuir isotherm (mg/g). Equation 10 can be used to calculate the Q_max_ using the Freundlich isotherm [44, 45].

10 $$Q_{\max } = k_{F} \left( {C_{o}^{1/n} } \right)$$

The equilibrium constants were presented in Table 3. The Freundlich isotherm provided poor fitting with low correlation coefficient values (R^2^) compared with the Langmuir isotherm. Consequently, the Langmuir isotherm may best describe the disposal of copper or cadmium ions using the Fe_3_O_4_/analcime nanocomposite. The greatest uptake capability of the Fe_3_O_4_/analcime nanocomposite toward the copper and cadmium ions is 176.68 and 203.67 mg/g, respectively.

**Table 3:** The equilibrium constants for the uptake of copper and cadmium ions by the Fe_3_O_4_/analcime nanocomposite

Metal ion	Langmuir			Freundlich		
	Q_max_ (mg/g)	K_L_ (L/mg)	R^2^	Q_max_ (mg/g)	K_F_ (mg/g)(L/mg)1/n	R^2^
Copper	176.68	0.6778	0.9998	199.46	100.31	0.7585
Cadmium	203.67	1.7662	0.9991	237.71	131.49	0.4995

The uptake capability of the Fe_3_O_4_/analcime nanocomposite was superior to that of many other adsorbents that published in the literature, as exhibited in Table 4.

**Table 4:** The uptake capability of some adsorbents toward copper and cadmium ions

Adsorbent	Metal ion	Uptake capability (mg/g)	Refs.
Aminated cellulose	Cu(II)	69.27	[31]
Aminated resin	Cu(II)	139.80	[32]
Chitin/chitosan-based aerogel	Cu(II)	59.21	[33]
Graphene oxide	Cu(II)	55.47	[34]
Carboxylated graphene oxide	Cu(II)	62.02	[34]
Alginate-halloysite	Cd(II)	88.00	[35]
Goethite-modified montmorillonite	Cd(II)	50.61	[36]
FAU zeolite	Cd(II)	74.07	[37]
Fe_3_O_4_/analcime nanocomposite	Cu(II)	176.68	This study
Fe_3_O_4_/analcime nanocomposite	Cd(II)	203.67	This study

#### Effect of regeneration and reusability

**Fig. 14:**
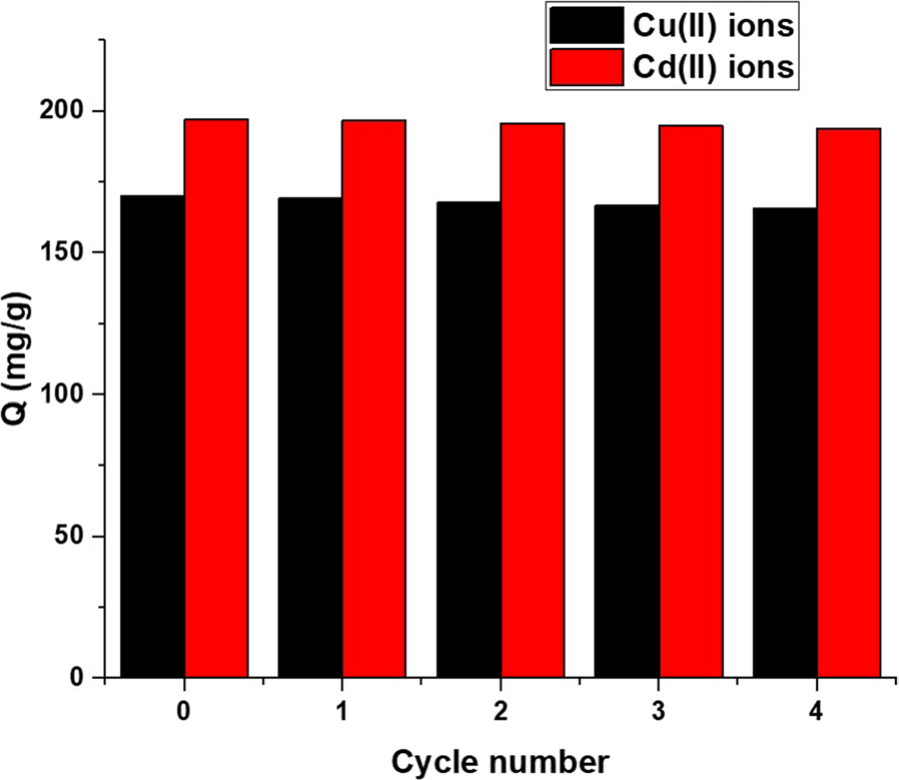
The effect of reusability for four consecutive cycles

Ethylenediaminetetraacetic acid disodium salt dihydrate is the head member of the family of ligands. Ethylenediaminetetraacetic acid disodium salt dihydrate is a hexadentate ligand forming highly stable five membered chelates with metal ions such as copper and cadmium ions. Hence, Ethylenediaminetetraacetic acid disodium salt dihydrate has the ability to completely desorb cadmium and copper ions from the surface of the nanocomposite. The uptake capability of the Fe_3_O_4_/analcime nanocomposite toward copper and cadmium ions did not change significantly for four consecutive cycles as shown in Fig. 14. Hence, the Fe_3_O_4_/analcime nanocomposite can be used many times without losing its effectiveness.

## Conclusion

The Fe_3_O_4_/analcime nanocomposite was facilely synthesized as novel nanostructures for the effective disposal of copper and cadmium ions from the aqueous solutions. The products were characterized using field emission scanning electron microscope (FE-SEM), Fourier transform infrared spectroscopy (FT-IR), and X-ray diffraction (XRD). The greatest uptake capability of the Fe_3_O_4_/analcime nanocomposite toward copper and cadmium ions is 176.68 and 203.67 mg/g, respectively.

## Data Availability

All data generated or analyzed during this study are included in this published article.

## References

[1] Xiang H, Min X, Tang CJ, Sillanpää M, Zhao F (2022). Recent advances in membrane filtration for heavy metal removal from wastewater: a mini review. J Water Process Eng.

[2] Zhang Y, Luo J, Zhang H, Li T, Xu H, Sun Y (2022). Synthesis and adsorption performance of three-dimensional gels assembled by carbon nanomaterials for heavy metal removal from water: a review. Sci Total Environ.

[3] Ayalew ZM, Guo X, Zhang X (2022). Synthesis and application of polyethyleneimine (PEI)-based composite/nanocomposite material for heavy metals removal from wastewater: a critical review. J Hazard Mater Adv.

[4] Mohan B, Kamboj A, Singh K, Singh G (2023). Metal-organic frameworks (MOFs) materials for pesticides, heavy metals, and drugs removal: environmental safety. Sep Purif Technol.

[5] Anastopoulos I, Ahmed MJ, Hummadi EH (2022). Eucalyptus-based materials as adsorbents for heavy metals and dyes removal from (waste)waters. J Mol Liq.

[6] De Beni E, Giurlani W, Fabbri L, Emanuele R, Santini S, Sarti C (2022). Graphene-based nanomaterials in the electroplating industry: a suitable choice for heavy metal removal from wastewater. Chemosphere.

[7] Yang X, Liu L, Wang Y, Lu T, Wang Z, Qiu G (2023). Sustainable and reagent-free cathodic precipitation for high-efficiency removal of heavy metals from soil leachate. Environ Pollut.

[8] Choumane R, Peulon S (2022). Innovative electrochemical process for a total removal and/or separation of soluble heavy metals. J Environ Chem Eng.

[9] Teng L, Yue C, Zhang G (2023). Epoxied SiO_2_ nanoparticles and polyethyleneimine (PEI) coated polyvinylidene fluoride (PVDF) membrane for improved oil water separation, anti-fouling, dye and heavy metal ions removal capabilities. J Colloid Interface Sci.

[10] Wang Q, Yu Z, Zhu X, Xiang Q, Chen H, Pang Y (2022). ZIF-67 modified MXene/sepiolite composite membrane for oil–water separation and heavy metal removal. J Ind Eng Chem.

[11] Huang H, Li Z, Wang H, Xia C, Yan P, Zhang Q (2022). Adsorption performance of layered double hydroxides for heavy metals removal in soil with the presence of microplastics. J Environ Chem Eng.

[12] Al-Wasidi AS, Naglah AM, Saad FA, Abdelrahman EA (2022). Modification of sodium aluminum silicate hydrate by thioglycolic acid as a new composite capable of removing and preconcentrating Pb(II), Cu(II), and Zn(II) ions from food and water samples. Arab J Chem.

[13] Al-Wasidi AS, AlSalem HS, Alshalawi AF, Naglah AM, Al-Anwar A, Abdelrahman EA (2022). Facile synthesis of a novel nanocomposite for determination of mercury and copper ions in food and water samples. Arab J Chem.

[14] Abdelrahman EA, Abou El-Reash YG, Youssef HM, Kotp YH, Hegazey RM (2021). Utilization of rice husk and waste aluminum cans for the synthesis of some nanosized zeolite, zeolite/zeolite, and geopolymer/zeolite products for the efficient removal of Co(II), Cu(II), and Zn(II) ions from aqueous media. J Hazard Mater.

[15] Abdelrahman EA, Hegazey RM, El-Azabawy RE (2019). Efficient removal of methylene blue dye from aqueous media using Fe/Si, Cr/Si, Ni/Si, and Zn/Si amorphous novel adsorbents. J Mater Res Technol.

[16] Abdelrahman EA, Hegazey RM (2019). Exploitation of Egyptian insecticide cans in the fabrication of Si/Fe nanostructures and their chitosan polymer composites for the removal of Ni(II), Cu(II), and Zn(II) ions from aqueous solutions. Compos Part B Eng.

[17] Abdelrahman EA, Hegazey RM (2019). Utilization of waste aluminum cans in the fabrication of hydroxysodalite nanoparticles and their chitosan biopolymer composites for the removal of Ni(II) and Pb(II) ions from aqueous solutions: kinetic, equilibrium, and reusability studies. Microchem J.

[18] Abdelrahman EA (2018). Synthesis of zeolite nanostructures from waste aluminum cans for efficient removal of malachite green dye from aqueous media. J Mol Liq.

[19] Mehdizadeh A, Najafi Moghadam P, Ehsanimehr S, Fareghi AR (2022). Preparation of a new magnetic nanocomposite for the removal of dye pollutions from aqueous solutions: synthesis and characterization. Mater Chem Horizons.

[20] Joseph J, Iftekhar S, Srivastava V, Fallah Z, Zare EN, Sillanpää M (2021). Iron-based metal-organic framework: synthesis, structure and current technologies for water reclamation with deep insight into framework integrity. Chemosphere.

[21] Hosseini J, Zare EN, Ajloo D (2019). Experimental and theoretical calculation investigation on effective adsorption of lead(II) onto poly(aniline-co-pyrrole) nanospheres. J Mol Liq.

[22] Bortolini HR, Lima DS, Perez-Lopez OW (2020). Hydrothermal synthesis of analcime without template. J Cryst Growth.

[23] Nassar MY, Abdelrahman EA (2017). Hydrothermal tuning of the morphology and crystallite size of zeolite nanostructures for simultaneous adsorption and photocatalytic degradation of methylene blue dye. J Mol Liq.

[24] Kassahun F, Taddesse AM, Teju E, Bogale Y (2023). Magnetic Al_2_O_3_/ZrO_2_/Fe_3_O_4_ nanocomposite: synthesis, characterization, and application for the adsorptive removal of nitrate from aqueous solution. Groundw Sustain Dev.

[25] Naini N, Sid Kalal H, Almasian MR, Niknafs D, Taghiof M, Hoveidi H (2022). Phosphine-functionalized Fe_3_O_4_/SiO_2_/composites as efficient magnetic nanoadsorbents for the removal of palladium ions from aqueous solution: kinetic, thermodynamic and isotherm studies. Mater Chem Phys.

[26] Kumar V, Kumar A, Chini MK, Satapathi S (2021). Fluorescent Fe_2_O_3_-CdSe nanocomposite probe for selective detection and removal of picric acid. Mater Chem Phys.

[27] Wang J, Zhang W, Wu H, Su F, Dai Q, Jiang Z (2023). Hydrothermal supramolecular preorganization synthesis of multi-morphological g-C_3_N_4_/Fe_2_O_3_ for photocatalytic removal of indoor formaldehyde under visible light. J Environ Chem Eng.

[28] Wang L, Zhang X, He J, Zhu J, Hu L (2022). The removal of ethyl mercaptan by Fe_2_O_3_/HNb_3_O_8_-NS composite. Inorg Chem Commun.

[29] Nabipour H, Rohani S, Batool S, Yusuff AS (2023). An overview of the use of water-stable metal-organic frameworks in the removal of cadmium ion. J Environ Chem Eng.

[30] Ferrah N, Abderrahim O, Didi MA, Villemin D (2011). Removal of copper ions from aqueous solutions by a new sorbent: polyethyleneiminemethylene phosphonic acid. Desalination.

[31] Pereira AR, Soares LC, Teodoro FS, Elias MMC, Ferreira GMD, Savedra RML (2020). Aminated cellulose as a versatile adsorbent for batch removal of As(V) and Cu(II) from mono- and multicomponent aqueous solutions. J Colloid Interface Sci.

[32] Niu L, Deng S, Yu G, Huang J (2010). Efficient removal of Cu(II), Pb(II), Cr(VI) and As(V) from aqueous solution using an aminated resin prepared by surface-initiated atom transfer radical polymerization. Chem Eng J.

[33] Kuang J, Cai T, Dai J, Yao L, Liu F, Liu Y (2023). High strength chitin/chitosan-based aerogel with 3D hierarchically macro-meso-microporous structure for high-efficiency adsorption of Cu(II) ions and Congo red. Int J Biol Macromol.

[34] Yang W, Cao M (2022). Study on the difference in adsorption performance of graphene oxide and carboxylated graphene oxide for Cu(II), Pb(II) respectively and mechanism analysis. Diam Relat Mater.

[35] Álvarez-Álvarez JA, Aguilar-Aguilar A, Robledo-Cabrera A, Ocampo-Perez R, Leyva-Ramos R, Padilla-Ortega E (2023). Contribution of halloysite as nanotubular clay mineral on mechanism and adsorption rate of Cd(II) onto nanocomposites alginate-halloysite. Environ Res.

[36] Zhao C, Yao J, Knudsen TŠ, Liu J, Zhu X, Ma B (2023). Performance and mechanisms for Cd(II) and As(III) simultaneous adsorption by goethite-loaded montmorillonite in aqueous solution and soil. J Environ Manag.

[37] Joseph IV, Tosheva L, Doyle AM (2020). Simultaneous removal of Cd(II), Co(II), Cu(II), Pb(II), and Zn(II) ions from aqueous solutions via adsorption on FAU-type zeolites prepared from coal fly ash. J Environ Chem Eng.

[38] Youssef HM, Shah RK, Algethami FK, Hegazey RM, Naglah AM, Al-Omar MA (2021). Facile hydrothermal procedure for the synthesis of sodium aluminum silicate hydrate/analcime and analcime for effective removal of manganese(II) ions from aqueous solutions. J Inorg Organomet Polym Mater.

[39] Kang YS, Risbud S, Rabolt JF, Stroeve P (1996). Synthesis and characterization of nanometer-size Fe_3_O_4_ and γ-Fe_2_O_3_ particles. Chem Mater.

[40] Khalifa ME, Abdelrahman EA, Hassanien MM, Ibrahim WA (2020). Application of mesoporous silica nanoparticles modified with dibenzoylmethane as a novel composite for efficient removal of Cd(II), Hg(II), and Cu(II) ions from aqueous media. J Inorg Organomet Polym Mater.

[41] Abdelrahman EA, Hegazey RM, Kotp YH, Alharbi A (2019). Spectrochimica acta part A : molecular and biomolecular spectroscopy facile synthesis of Fe_2_O_3_ nanoparticles from Egyptian insecticide cans for ef fi cient photocatalytic degradation of methylene blue and crystal violet dyes. Spectrochim Acta Part A Mol Biomol Spectrosc.

[42] Ubhi MK, Kaur M, Singh D, Greneche JM (2017). Nanocomposite of γ-Fe_2_O_3_ immobilized on graphene oxide for remediation of Ni(II) ions: kinetics, isotherm and thermodynamics studies. Process Appl Ceram.

[43] Kaur J, Kaur M, Ubhi MK, Kaur N, Greneche JM (2021). Composition optimization of activated carbon-iron oxide nanocomposite for effective removal of Cr(VI)ions. Mater Chem Phys.

[44] Al-Wasidi AS, Maram W, Reem TB, Eida MA, Al-Farraj S, Abdelrahman EA (2023). Facile synthesis and characterization of sodium magnesium silicate hydrate/sodium magnesium silicate hydroxide as novel nanostructures for the efficient removal of methylene blue dye from aqueous media. J Inorg Organomet Polym Mater.

[45] Al-Wasidi AS, Basha MT, Alghanmi RM, Al-Farraj ES, Abdelrahman EA (2023). Functionalization of sodium magnesium silicate hydroxide/sodium magnesium silicate hydrate nanostructures using 2, 3-dihydroxybenzaldehyde as a novel nanocomposite for the efficient removal of Cd(II) and Cu(II) ions from aqueous media. Separations.

